# Learning health systems to implement chronic disease prevention programs: A novel framework and perspectives from an Australian health service

**DOI:** 10.1002/lrh2.10466

**Published:** 2024-10-15

**Authors:** Luke Wolfenden, John Wiggers, Courtney Barnes, Cassandra Lane, Daniel Groombridge, Katie Robertson, Jannah Jones, Sam McCrabb, Rebecca K. Hodder, Adam Shoesmith, Nayerra Hudson, Nicole McCarthy, Melanie Kingsland, Emma Doherty, Emily Princehorn, Meghan Finch, Nicole Nathan, Rachel Sutherland

**Affiliations:** ^1^ Hunter New England Population Health Hunter New England Local Health District Newcastle NSW Australia; ^2^ School of Medicine and Public Health The University of Newcastle Newcastle NSW Australia; ^3^ National Centre of Implementation Science The University of Newcastle Newcastle NSW Australia; ^4^ Hunter Medical Research Institute New Lambton Heights NSW Australia

**Keywords:** chronic disease, implementation, learning health system, prevention, public health

## Abstract

**Background:**

Chronic diseases are a considerable burden to health systems, communities, and patients. Much of this burden, however, could be prevented if interventions effective in reducing chronic disease risks were routinely implemented.

**Aims:**

The aim of this paper is to discuss the role of public health agencies in preventing chronic disease through the application of learning health system (LHS) approaches to improve the implementation of evidence‐based interventions.

**Materials and Methods:**

We draw on the literature and our experience operating a local LHS in Australia that has achieved rapid improvements in the implementation of chronic disease prevention interventions.

**Results:**

The proposed LHS framework has been adapted to be both implementation and chronic disease prevention focused. The framework describes both broad improvement processes, and the infrastructure and other support (pillars) recommended to support its core functions.

**Conclusion:**

The framework serves as a basis for further exploration of the potentially transformative role LHS's may have in addressing the chronic disease health crisis.

## 1 INTRODUCTION

Healthcare costs as a percentage of gross domestic product are projected to double between 2009 and 2040 in the United States.^1^ These increases are largely driven by the escalating treatment costs associated with chronic diseases, such as cancer and heart disease.^2^ Much of this disease burden is preventable. For example, half of all cancers could be prevented if interventions already known to be effective in reducing cancer risk were adequately implemented.^3^, ^4^ The delivery of effective interventions, particularly at the scale required to achieve population‐wide reductions in chronic disease risk, remains an unresolved challenge.^5^, ^6^, ^7^

While comprehensive and multi‐sectoral approaches are recommended for the prevention of chronic disease, in most countries, it is the primary responsibility of public health agencies, such as national/state departments of health, non‐government organizations, and local health services (hereafter referred to as “prevention agencies”^8^). These agencies operate at different levels (macro, meso, and local) to support the health of populations through policies, resourcing, infrastructure, or service delivery.^8^, ^9^ They may directly deliver (or implement) prevention services or support the implementation of health promoting policies and programs by other organizations in the community (e.g., schools). Prevention agencies operating at different levels of the prevention systems have different opportunities (or mechanisms) to influence the implementation of chronic disease prevention interventions (Table 1—see also Supplementary Figure 1).

**TABLE 1:** Examples of agencies involved in prevention of chronic disease.

Level	Examples of prevention agencies and other organizations	Potential mechanisms of influence
Macro	Government departments, regulatory authorities, or statutory bodies	Policy, financing, regulation and enforcement, monitoring etc.
Meso	Non‐government organizations, professional associations, training or accreditation organizations, health services	Advocacy, setting professional standard and capacity building, audit and feedback etc.
Local	Targeted community settings (e.g., schools), local government, community groups	Local needs assessments, priority setting, organizational processes etc.

The limited success of many large‐scale prevention initiatives in reducing chronic disease risks has been partly attributed to ineffective implementation.^5^, ^6^, ^7^ As a consequence, reviews of the impact of national chronic disease prevention strategies (e.g., Australian National Chronic Disease Strategy) recommend the use of “evidence‐informed implementation strategies” to address this policy‐to‐practice gap.^10^ Implementation strategies are methods or techniques used to promote the adoption and implementation of evidence‐based programs, policies, and practices.^11^ Training, audit and feedback, regulations, or financing are examples of implementation strategies. Despite their importance, research addressing the effectiveness of such strategies has not been a focus of prevention research.^12^, ^13^, ^14^ Further, implementation science has been criticized for being inaccessible and not well aligned to the needs, priorities, and timelines of organizations and professionals responsible for program implementation.^15^, ^16^, ^17^ Such issues are symptomatic of a system of evidence generation that is, in part, divorced from the process of its application.

### 1.1 Learning health systems to enhance the implementation of chronic disease prevention interventions

A paradigm shift is required in how evidence is efficiently produced and applied to better implement chronic disease prevention interventions. To improve healthcare more broadly, the Agency for Healthcare Research and Quality and the Institute of Medicine recommend the re‐orientation of health services toward “learning health system” (LHSs).^18^, ^19^ LHSs use data‐driven approaches to efficiently build knowledge for health system improvement by continuously undertaking research with and within the health system. Within a LHS, health services recognize key knowledge gaps, integrate research methods (e.g., randomized trials) into their “usual practice” to address them, and use the findings to continuously improve care and patient outcomes. This integrated process can eliminate the “translation lag” as research is conducted in contexts where outcomes are immediately applied. LHSs are a pathway to quality improvement,^20^ are systematic, and can be undertaken at different scales (from local health services to international health systems).^21^ They are also characterized by their integration of internal data and experience with external evidence, use of learning communities, data platforms, and informatics.^20^ While an emerging model of knowledge creation and application, LHSs have demonstrated rapid advancements in healthcare, achieving improvements in clinical practice, reductions in healthcare costs, and patient mortality.^22^, ^23^

LHSs may be a transformative approach to generating and applying evidence that enhances the effective and timely implementation of chronic disease prevention programs. First, it represents a significant departure from current approaches to such enhancement which follow a fixed, linear approach that is not characterized by data‐driven improvement cycles. For example, reviews have identified 30 trials testing strategies to implement prevention interventions in childcare services, sporting clubs, and workplaces^24^, ^25^, ^26^ but none included explicit processes of sequential (or ongoing) testing and adaptation of strategies to improve their effects. Second, the approach is aligned to contemporary perspectives in implementation science, such as the dynamic sustainability framework which emphasizes continuous learning, ongoing adaptation, and improvement of interventions.^27^ Third, the involvement of prevention agencies in generating evidence ensures the local relevance of the knowledge created to local decision‐making and improves the relevance of evidence for prevention policy and practice decision making more broadly. As such, the National Institute of Health Consortium of Cancer Implementation Science has established action groups dedicated to advancing LHSs in prevention agencies.^28^

While LHSs may be a promising approach to prevent chronic disease, the LHS literature and guidance to date have focused almost exclusively on its application to improve clinical‐based patient‐focused health care.^23^ However, the public health prevention system differs from clinical health care in a number of ways. First, it comprised different actors (e.g., prevention agencies, community organizations) than those of clinical medicine. Second, it is population rather than individual patient focused, and hence requires different capabilities, infrastructure, and resources to support the implementation of effective interventions. Third, it is focused on improving the implementation of health programs or policies, rather than the development of more effective drugs, devices, procedures, or therapies. As a consequence, a LHS approach to improve the implementation of chronic disease prevention interventions requires different attributes. As such, LHS models, guidance, and frameworks aimed at improving the implementation of chronic disease prevention programs by prevention agencies tailored to these unique needs are required.

The purpose of this commentary is to discuss what such a LHS may look like, its key processes and attributes. We present a LHS framework specifically designed for the implementation of chronic disease prevention interventions by public health prevention agencies, the first, we believe to do so. The framework is presented to facilitate further conversation, innovation, and research regarding how LHSs can better prevent the burden of chronic diseases to individuals, communities, and health systems.

## 2 A NOVEL FRAMEWORK FOR A LHS TAILORED TO THE PREVENTION OF CHRONIC DISEASE BY A PREVENTION AGENCY

**FIGURE 1:**
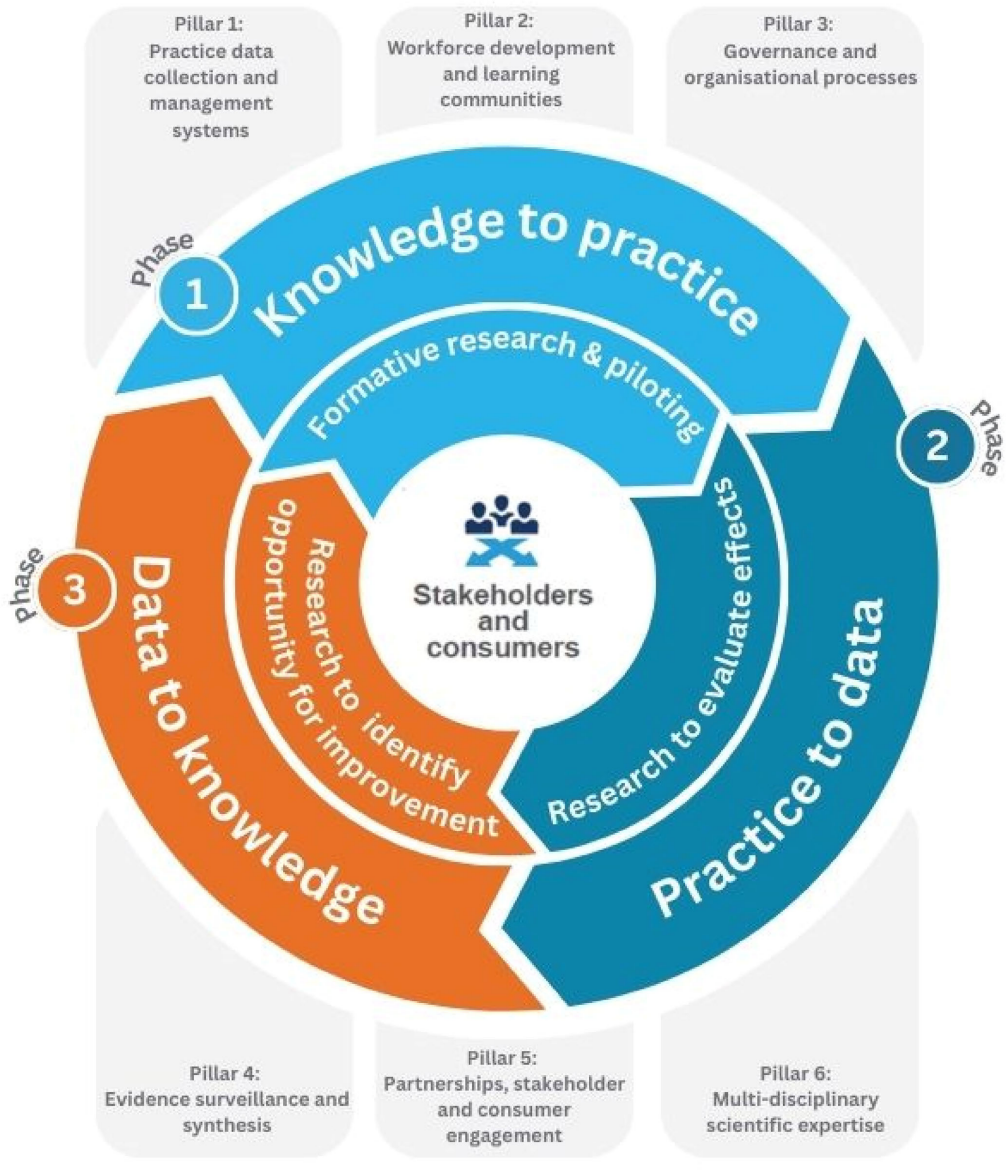
The proposed learning health system framework for prevention.

We have applied LHS principles in a public health unit (Hunter New England Population Health) serving a Local Health District in New South Wales (NSW), Australia.^29^ We have demonstrated that doing so can yield significant improvements in the implementation of chronic disease prevention programs by community organizations and reduce the costs of implementing such programs.^29^, ^30^, ^31^, ^32^, ^33^ For example, using sequential randomized trials, in just 3 years, we developed strategies that improved school compliance with a school nutrition policy from 5% to 60%, and reduced the costs of achieving this by as much as 55%.^32^ In this context, we offer our reflections on the core functions of a prevention agency LHS. To do so, we present a LHS framework for prevention to improve the implementation of chronic disease prevention programs (Figure 1). The framework was also informed by the Learning Healthcare Project,^34^ a review of LHS frameworks,^35^ recommendations for LHSs with an implementation focus,^36^ and to better address the prevention of chronic disease. This novel LHS framework tailored to improve the implementation of prevention programs consists of three core phases that describe the LHS processes of improvement; and six supports, or “pillars” that support the functioning of the LHS.

### 2.1 LHS phases

LHSs undertake data‐driven improvement processes. Broadly, these processes fall into three phases. All phases draw on the insight from prevention agency staff, researchers, consumers, and other stakeholders—offering perspectives critical in co‐designing contextually relevant approaches to implementing prevention interventions. Improvement phases may continue until implementation has been sufficiently optimized or sustained in routine service delivery—that is, the point at which further efforts to enhance implementation are considered unlikely to achieve a level of improvement warranting the required investment.^37^

During the “knowledge to practice” phase (phase 1), prevention agencies or other organizations seeking to improve implementation apply insights gleaned from various data sources to improve practice. This may include changes to models of care, the provision of additional professional development, or the introduction of decision support tools. Evidence generation in this phase, therefore, is focused on formative evaluation and piloting to appraise and assess planned improvements to the implementation or prevention programs.^38^ This could, for example, include research to assess the acceptability of interventions or implementation strategies, exploring adaptations to these to better fit local context and resources, exploring barriers and facilitators (determinants) to implementation, and piloting the feasibility of new or amended implementation strategies.

During the “practice to data” phase (phase 2), data is collected to evaluate or examine the effects of strategies enacted to improve the implementation of chronic disease prevention programs, policies, or practices. Given the latency between exposure to a chronic disease prevention program and chronic disease onset, assessments of program effectiveness usually involve measures of more proximal outcomes, such as changes to chronic disease risk factors (e.g., tobacco use). As public health services should be seeking to implement interventions known to be effective in addressing chronic disease (or its risk factors), evaluation of strategies to improve implementation of such intervention typically focus on measures of the quality or fidelity of their delivery (implementation). Surveys of public health policymakers and practitioners in Australia suggest data regarding the effectiveness, equity impacts, acceptability, and feasibility of implementation efforts are important to guide their improvement decisions.^39^ Similar outcomes are also recommended for implementation trials more broadly.^40^, ^41^ A range of research designs can be embedded in the routine service delivery of prevention agencies to assess the outcomes of efforts to improve implementation. Within our public health unit, we have embedded sequential randomized controlled trials (RCTs) to support rapid improvements in the implementation of chronic disease prevention programs.^29^, ^32^, ^33^, ^42^ However, we acknowledge such designs may not be feasible or appropriate for the evaluation of some implementation strategies (e.g., policy, regulatory, or financing strategies). The collection of qualitative data is also important to describe the implementation context, enhance understanding of implementation outcomes, and/or identify areas to target change. Its routine integration into processes of care has been described as the next “paradigm” of health care.^43^

In the “data to knowledge” phase (phase 3), data is analyzed, synthesized, and interpreted to identify opportunities for ongoing improvement. This typically involves drawing together different forms of data to elucidate substantive meaning, such as data from local evaluations, evidence syntheses (e.g., reviews), implementation costings, and qualitative insights. Such data should be used to assess whether a strategy to implement an intervention had beneficial (and no substantive adverse) effects, for whom it was beneficial (was it equitable), how it worked (causal mechanisms), how much it cost, and the experience of key stakeholders and consumers.^40^ Such evidence is used in this phase to improve implementation efforts, for example by adding, modifying, or removing components of an implementation strategy to improve its effectiveness, efficiency, equity, acceptability, sustainability, or cost‐effectiveness. Given the complexity of (preventive) health systems, there is often uncertainty regarding the most effective means of improving implementation, even when data collection is comprehensive.^44^ Improvement decisions, therefore, typically require substantive judgment. Decision‐makers must be assured that there is sufficient evidence to indicate that the quality of implementation is insufficient and that meaningful improvements can be anticipated from any modifications. We have found co‐design processes involving multiple perspectives to interpret available data may overcome such challenges and support iterative data‐driven, value‐based decision‐making. Additionally, tools, such as the GRADE Evidence to Decision Framework^45^ and the WHO INTEGRATE framework,^46^ can assist in making informed decisions.

### 2.2 LHS support (pillars)

The iterative and LHS improvement phases described above require supportive infrastructure, expertise, and resources. Similar to that of Menear et al.'s^47^ conceptual framework for value‐creating LHS, we define six LHS support pillars, considered important for well‐functioning LHSs. Here, we describe how these can support the implementation of interventions to prevent chronic disease.

#### 2.2.1 Pillar 1: Practice data collection and management systems

The National Institute of Medicine considers routine collection of valid, relevant, and usable practice data as foundational for “rapid‐learning healthcare.”^48^ In clinical settings, there has been significant investment in electronic medical record systems to serve this function. A lack of such systems, however, is a fundamental constraint to data‐driven improvement in community settings targeting prevention of chronic diseases.^49^ Although regulatory authorities or accreditation agencies collect data on the implementation of some health‐related programs, policies, or practices (e.g., food safety) in some settings, it is typically inaccessible to external agencies, not collected with sufficient frequency, or is insufficient to support improvement. Rather, improvement requires data which identifies evidence‐practice gaps (where implementation support is required and for whom), barriers and facilitators to implementation (to design effective implementation support strategies and required for tailoring to specific population groups), and monitor changes in practice over time (e.g., following execution of implementation strategies) and to examine their impacts universally and in priority populations (to assess equity outcomes).

We are aware of few systems designed to do so for the implementation of prevention programs in non‐clinical community settings. In NSW, Australia, the Population Health Implementation Management System provides a system for staff of local health promotion units to routinely record the strategies (e.g., training, local facilitation, provision of resources) provided for implementation of healthy eating policies and practices in childcare services and schools.^50^ The data are used by health promotion teams to target services that require additional support and to evaluate and improve the impacts of implementation support provided. The data are also used by the NSW Ministry of Health, to set and track health promotion unit achievement of implementation key performance indicators. The system is credited with supporting the successful scale up of health promoting interventions in these settings and reducing the prevalence of child obesity.^51^ In the absence of such systems, purposeful data collection efforts that are low cost and provide implementation as close as possible to real time may be required. For example, website data scraping, geocoding, and other technologies are being used to assess the healthiness of food retail environments (e.g., healthy food availability, food pricing, and promotion).^52^, ^53^, ^54^ Our public health unit established a call centre to undertaken surveys of community organizations (e.g., schools) to monitor implementation of health promotion initiatives it is responsible for supporting.^29^ Data collection systems require secure data management and trial infrastructure, typically provided via partnerships with medical research institutes and universities as well as processes to ensure data privacy, sovereignty, and ethical use.

#### 2.2.2 Pillar 2: Professional workforce development, and learning communities

LHSs require public health practitioners to be competent in supporting the implementation of evidence‐based policies and programs and also capable of using data for improvement.^49^ The chronic disease prevention workforce is inherently multi‐disciplinary and has a long history of using data to set priorities and guide practice. They also have expertise in engaging stakeholders and communities, and skills in research and evaluation methods including evidence synthesis, epidemiology, and statistics.^55^ There are, however, many areas where professional development seems warranted.^56^, ^57^ For example, Leppin et al.'s^58^ framework for “fostering scientific and practical capacity for implementation and increasing the routine application of research knowledge in real‐world systems” is recommended for LHSs. It emphasizes a range of implementation skills required for a workforce capable of executing a LHS, including expertise in adapting interventions to context, identifying implementation determinants, stakeholder engagement, and research‐practice partnerships.^58^ Surveys of Australian chronic disease prevention practitioners we have undertaken suggest these as areas where greater professional development is required.^59^, ^60^ Internationally, public health practitioners also reportedly lack knowledge in the application of implementation science tools and techniques.^57^ Such findings suggest updating existing competencies for the public health and prevention workforce to align with the expertise required for LHS will be important if LHS are to be a widespread model of preventing chronic disease.^61^

While a range of formal training to support workforce development in these areas are available, the use of more applied and integrated approaches is recommended. Learning communities have been described as foundational for LHSs.^20^ They comprise a group of collaborators seeking to address an agreed health system challenge. They provide a forum for knowledge exchange between collaborators, often including researchers and prevention policymakers and practitioners.^62^ We have implemented a state‐wide community of practice as part of a LHS whereby public health units and implementation scientists exchange knowledge, data, and learnings to implement health promotion programs.^63^ Other workforce development strategies have also been suggested to support the operation of LHS.^20^ For example, our public health unit has invested in the “embedding” of researchers with dual responsibilities (prevention program implementation and research) in the agency. This “academic practitioner” model ensures those in key leadership positions have the breadth of skills necessary to support the generation and application of evidence for public health improvement.^29^ It also ensures that scientific expertise, such as for undertaking or interpreting evidence synthesis, research, and analyses, is available within agency where improvements are sought.

#### 2.2.3 Pillar 3: Governance and organizational processes

Operation of LHSs require strong governance and organizational processes to ensure proper execution of its core functions to achieve LHS goals.^20^ Effective governance structures and organizational processes provide a mechanism to define the LHS purpose, priorities, and scope, and establish operational standards. They are also a mechanism for defining contributor roles, decision‐making processes, and ensuring transparency and accountability for all engaged parties.^20^, ^64^ This is particularly critical for public health oriented LHSs involving a large number of diverse stakeholders that require consideration of regulations or institutional mandates, or differences in professional or organizational culture, values, or interests that may impede collaborative activity.^47^

An important function of governance and organizational processes are to support collaboration and integration of LHS partners, as well as data, expertise, and resources. Structural barriers to inter‐organizational collaboration can often be managed through formal agreements, regulations, or financial support (contracts) or other processes. Those related to values or culture require strong leaderships, communication, shared goals, and the development of trust.^8^ Other organizational processes recommended for LHSs are policies and strategies to ensure the financial sustainability of the LHS and that govern resource use and allocation.^47^ The use of incentive and recognition systems has also been suggested to foster a support for and a culture of continuous improvement.^20^

#### 2.2.4 Pillar 4: Evidence surveillance, and synthesis

Improvement decisions of LHS require consideration of up‐to‐date evidence syntheses to contextualize local research findings with the broader evidence base. Databases of high‐quality systematic reviews of public health interventions (and implementation strategies), such as those hosted by Cochrane, or Health Evidence (Canada),^65^ provide important evidence infrastructure for public health oriented LHSs. The conduct of systematic reviews of the effectiveness of strategies to improve the implementation of chronic disease prevention intervention was a common, though time consuming activity undertake in our Australian LHS. Recent innovations in systematic review technologies, such as text mining, crowd sourcing, and artificial intelligence, have enabled the production of “living reviews.” In such reviews, evidence surveillance is ongoing, enabling inclusions of research into evidence syntheses to guide decision‐making as soon as it is available.^66^, ^67^ Such reviews are particularly useful resources for LHSs as they ensure decision‐making considers the best available evidence at the time. Advancements in technology, including generative artificial intelligence, will no doubt reduce the time and resource requirements of undertaking evidence reviews. It may also identify new opportunities for the surveillance of public health and implementation science literature. LHSs should invest in these technologies to help inform improvements.

#### 2.2.5 Pillar 5: Partnerships, stakeholder, and consumer engagement

The formation of strategic partnerships with organizations with specialist or complimentary expertise is an important part of LHSs given the diverse skills and expertise required for their operation. Fostering partnerships with organizations across different levels of the prevention system who can support the implementation of programs may also yield more effective and enduring implementation. While prevention‐focused LHSs primarily target the actions of organizations (e.g., hospitals, schools) and their staff (e.g., clinicians, teachers) to facilitate the delivery of interventions, community members (consumers) and other stakeholders affected by these provide important guidance about what and how best prevention programs are implemented.

Guidance to support the identification of relevant stakeholder^68^ and appropriate consumers relevant to chronic disease prevention has been published elsewhere.^69^ Their engagement is important to ensure LHSs improve health.^70^ This could be achieved through inclusive decision‐making processes, and representation of key stakeholders and consumers in governance structures. Co‐design and co‐production processes are commonly used in public health and implementation science to engage key stakeholders and consumers.^71^ They too are foundational for LHSs.

Health equity is a core public health value.^72^ Engagement of those representing priority populations (e.g., those experiencing disadvantage) can help ensure LHSs do not exacerbate existing health inequities. While achieving equity in public health remains a considerable challenge,^72^ we believe implementation‐focused LHSs may be well placed to help address this. In Australia, our local public health unit executed a range of innovations in recruitment, shared decision‐making, and other processes to support the more equitable and culturally appropriate provision and implementation of chronic disease prevention programs.^70^ While our experience of this process is that it has yielded many benefits for communities, it has also highlighted the considerable progress yet to be made.

#### 2.2.6 Pillar 6: Multi‐disciplinary scientific expertise

LHSs are a departure from conventional, linear, approaches of implementing public health interventions that are not characterized by ongoing testing, refinement, and improvement.^73^ Instead, they seek to identify and apply strategies that are system‐based, data‐driven, context‐specific, and evolve based on evaluation and feedback. This requires the integration of science from a range of disciplines and inclusive of qualitative and quantitative methods. We argue those are primarily (i) systems science, (ii) implementation science, and (iii) data science including biostatistics, evidence synthesis, and informatics. Systems science perspectives provide a holistic view of health, aiding an understanding of complexity and how factors operating at different levels may interact to influence health systems and their outcomes.^74^ Implementation science provides methods to help identify public health interventions amenable to implementation, factors that may impede or promote their delivery, and strategies to achieve successful implementation. Data‐science and informatics enables real‐time processing and analysis of information to inform decision making, enabling adaptive approaches to improve implementation that is responsive to learning and changing contexts.

While a LHS provides a framework that can bring these disciplines together, doing so, remains a challenge.^75^ It will require the strategic recruitment, and/or partnership with individuals or organizations which bring such scientific expertise. It also represents a new way of working for academics and public health researchers. The Agency for Healthcare Research and Quality “LHS Researcher Core Competencies” has been published to support the development of training programs to advance these research skills.^76^ It includes competency domains relevant to systems science, improvement and implementation science, and informatics. However, the integration of multi‐disciplinary teams also necessitates a culture of collaboration and teamwork, founded on mutual understanding and respect, effective communication, shared goals, and structured decision‐making. Our experience in Australia is this can be fostered through embedding researchers in health services. A recently published framework for overcoming challenges to teamwork in healthcare can also help organizations support productive and collaborative teams.^77^ We believe it will also be a useful resource for supporting multidisciplinary teams for a LHS for prevention.

## 3 OPERATIONALIZING A LHS

The lack of limited application of LHSs in public health and chronic disease prevention likely reflects the significant challenges in operationalizing them. LHS represent a complete alignment of research and health system enterprises. Reorienting existing public health agencies to effectively generate and apply evidence for better implementation of public health services will require strong leadership and considerable resources. For many prevention agencies, these essential components needed to operate as a LHS may not materialize, particularly in the short term, due to competing demands, workforce limitations, and fiscal constraints of the health system.^78^ Nonetheless, even incremental investments to improve research use in decision making are likely to enhance the impact of public health services. Supplementary file 2 outlines a range of strategies that public health agencies can employ to engage in implementation research. Developed following an evidence review and policy dialogue with chronic disease prevention agencies in Australia, it provides guidance for agencies ranging from those looking to engage external academic agencies to undertake research to inform their decision making to those aiming to lead their own research agenda to improve their services (akin to a LHS).^9^ Furthermore, LHSs may not need to be ubiquitous to have substantive public health and health system impacts. For example, evidence generated by LHSs addressing priority health system challenges can be applied to improve services by other prevention agencies.

To support the development and operation of LHSs focused on improving the implementation of chronic disease prevention programs, we provide two case studies illustrating the application of the LHS framework: one operationalized at a local organizational level and the other extended across the state of NSW.

### 3.1 Case study 1: Local‐level LHS approach to improve school implementation of a physical activity policy

Hunter New‐England Population Health (HNEPH) is a government funded population health unit responsible for delivering health promotion services to a diverse region with a population of nearly 1 million people. Its primary role is to support organizations, such as hospitals and schools, to implement evidence‐based interventions, including those to reduce chronic disease risk. Since 2005, HNEPH has partnered with the University of Newcastle to embed researchers into its service delivery team. This collaboration operates within a unified governance structure that is responsible for co‐designing and co‐producing both services and research.^29^ HNEPH initiatives are evaluated and produce research outputs, and all research within the unit is undertaken to improve the impact of its services. HNEPH approach involves a range of strategies to support the integration of research into its health promotion services, including senior leadership with dual academic (University) and HNEPH appointments, access to university research infrastructure (e.g., Library, PhD scholarships, data platform) and external grants for service aligned research, incorporating research and evaluation as core competencies in HNEPH staff position descriptions and appraised in performance reviews, and facilitating knowledge exchange via “in‐house” training, and joint (academic/service) student supervision models.^33^ Further information about the operation of this “embedded” model of research and practice is described in detail elsewhere.^29^, ^33^

As part of its investments in chronic disease prevention, HNEPH aimed to improve the implementation of the NSW Department of Education School Sport and Physical Activity Policy which mandated the minimum period of time schools schedule organized physical activities for students across the week (150 min).^79^ Such policies are known to be effective in increasing student physical activity when implemented.^80^ However, 2 years following the policy's release, local schools were scheduling only approximately 112 min of organized activity per week, significantly less than the recommended amount.^81^ At that time, implementation research had not provided sufficient evidence to guide how best this could be addressed.^82^

Drawing on LHS infrastructure and processes (Table 2), HNEPH conducted a series of improvement cycles, where tacit knowledge, local research and data, and review evidence were used to understand determinants of policy implementation and design strategies to improve the time school's scheduled for physical activity (knowledge to practice phase). A RCT was undertaken to test the extent to which the strategies employed by HNEPH improved policy implementation by schools (practice to data phase). Data were analyzed and interpreted by HNEPH staff and stakeholders to identify opportunities for further improvement (data to knowledge phase).^33^ HNEPH aimed to optimize its strategy for improving school implementation of the policy within its resource constraints and in a manner acceptable to schools.

**TABLE 2:** Two examples of how the learning health system has been operationalized.

Pillars	Case study 1	Case study 2
*Pillar 1: Practice data collection and management systems*	A project specific data collection system was developed and jointly funded by HNEPH and researcher‐secured (University) income to assess key implementation outcomes (policy adoption), processes, and costs Data management and randomized group allocation were conducted by academic staff embedded within HNEPH, utilizing the data infrastructure provided by the University	A project specific electronic data collection system was developed by NCOIS to assess key implementation outcomes (e.g., program adoption) and processes Data collection, management, and randomized group allocation were centralized and undertaken by NCOIS researchers
*Pillar 2: Professional workforce development, and learning communities*	Embedded behavioral and implementation scientists from HNEPH applied evidence‐based, theoretical, and evaluation frameworks to guide the development, evaluation, and refinement of the strategy. This work was carried out in partnership with HNEPH staff, who brought valuable experience from working with schools and local communities. HNEPH staff were also responsible for executing the implementation strategy To support learning, HNEPH staff and embedded researchers formed a single, integrated team responsible for the operational management of the project (implementation and evaluation). The team met regularly to reflect on project progress, share experiences and learnings, and engage in collaborative decision making	NCOIS researchers provided training and facilitated co‐design workshops for health promotion unit staff, assisting them in applying evidence and implementation frameworks to develop local implementation strategies. Health promotion staff, in turn, engaged with other local stakeholders as appropriate All local health promotion units participated in a community of practice, fostering knowledge exchange, group learning, and sharing findings from local initiatives. This collaborative environment helped identify opportunities for further strategy improvement
*Pillar 3: Governance and organizational processes*	HNEPH was ultimately responsible for decisions regarding the support provided to facilitate schools' implementation of the physical activity policy HNEPH operates with an integrated governance structure, ensuring coordinate efforts across stakeholders An Advisory Group, with representation from stakeholder groups, was formed to provide guidance throughout the project Stakeholder roles, including the execution of specific implementation support strategies, were clearly defined and documented in partnership agreements Deliberative decision‐making processes regarding “improvements” to the policy implementation support strategies (such as adding, discarding, or modifying elements) were informed by local research findings, the broader evidence base (evidence synthesis), and the tacit knowledge and expertise of Advisory Group members	Each health promotion unit was responsible for decisions about the support provided to facilitate school adoption of SWAP‐IT within their jurisdiction A collective governance structure provided oversight for the project, with representation from each health promotion unit and NCOIS researchers Health promotion unit teams executed implementation support strategies via the governance and processes established within their organization NCOIS offered centralized research and evaluation support to all health promotion units, conducting data collection and analysis, and providing evidence to inform health promotion unit decisions regarding strategy improvement
*Pillar 4: Evidence surveillance, and synthesis*	Systematic reviews on the effectiveness of strategies to implement school‐based policies, and of implementation barriers, were undertaken and updated throughout the project The findings from these reviews were integral to informing improvement decisions, ensuring they were guided by the best available evidence internationally in conjunction with data from local evaluations	Systematic reviews on the effectiveness of strategies to implement school‐based policies and programs were undertaken and updated throughout the project period by NCOIS researchers The findings from these reviews were a key input in the strategy co‐design workshops Reviews and findings from trials of strategies implemented by each health promotion unit informed decisions for strategy improvement within these units
*Pillar 5: Partnerships, stakeholder and consumer engagement*	HNEPH established partnerships with key policy agencies (Department of Education; Ministry of Health), as well as researchers, other health services, and local school principals and teachers Partners were actively engaged in the project's research co‐design, co‐production, decision‐making, and research dissemination via the Advisory group, task specific working groups, partnership agreements, and other processes	The primary partnership for the initiative was between NCOIS and the health promotion units, focusing on co‐design, research co‐production, decision making, and dissemination Local partnerships were formed by local health promotion units as needed and could include representatives from the education or health sectors, priority populations, local school staff, and parents
*Pillar 6: Multi‐disciplinary scientific expertise*.	Researchers embedded within HNEPH and engaged in the projects were multi‐disciplinary, encompassing a wide range of scientific skills (e.g., evidence synthesis, biostatistics, clinical trials) and expertise (e.g., behavioral and implementation science) Additional expertise, such as health economics, was secured through external contracts to support specific aspects of the project	The multi‐disciplinary expertise of NCOIS researchers provided necessary scientific skills (e.g., evidence synthesis, biostatistics, clinical trials) and expertise (e.g., behavioral and implementation science) for the project

Abbreviations: HNEPH, Hunter New England Population Health; NCOIS, National Centre of Implementation Science.

To inform decision‐making, each RCT assessed the impact of HNEPH support on policy implementation (minutes of scheduled physical activity), implementation costs (from HNEPH perspective), and processes measures such as acceptability. In an initial RCT, strategies employed by HNEPH (e.g., school champions for change and education outreach visits) enhanced scheduled physical activity by 36.6 min.^83^ Modifications to these strategies, tested in a second RCT, further enhanced this effect to 44.2 min.^81^ The focus of the third improvement cycle was to maintain these effects while reducing implementation strategy costs for HNEPH, primarily by changing the modality of providing school support (e.g., telephone support in place of face‐to‐face). Accordingly, in the third improvement cycle, a noninferiority RCT found that the adapted strategies had a similar effect to the strategies that achieved a 44‐min improvement in policy implementation (96% probability of non‐inferiority) but reduced HNEPH support costs from $1057 to $648 per school.^84^

### 3.2 Case study 2: System‐level LHS to improve implementation of a healthy school lunchbox program

In this case study, we describe how our LHS has been extended from HNEPH, to a network of health promotion units across NSW. Funded by the NSW Ministry of Health, these units address state health priorities within their local districts. Despite addressing similar health issues through similar interventions, these units operate in diverse contexts (e.g., metropolitan vs. remote) and have varying capacities for implementation support. This includes limited access to data infrastructure or expertise for generating and applying research evidence. As such, the approaches employed by many health promotion units are not formally assessed for their effectiveness. A LHS applied at a system level provided an opportunity to leverage and learn from diverse implementation approaches across health promotion units. By systematically assessing and sharing innovations in implementation, such a system can support and enhance the effectiveness of all health promotion strategies statewide.

The HNEPH LHS framework was applied to improve the implementation of a healthy school lunchbox program called “Swap‐it” (Table 2). Swap‐it has shown to be effective in improving student weight status and dietary intake.^42^, ^85^ Health promotion units identified improving the nutritional quality of foods packed for children and brought from home as a priority. The National Centre of Implementation Science (NCOIS) at the University of Newcastle provided centralized scientific expertise and infrastructure to support the LHS.^86^ Funded by a National Health and Medical Research Council grant, NCOIS was tasked with supporting knowledge translation and is led by the architects of the LHS framework developed and applied by HNEPH. NCOIS assisted health promotion units through the framework's improvement cycle process, including the development of locally tailored strategies to support the adoption of Swap‐it and the integration of RCTs into these efforts (see Table 2). These trials were harmonized across the network, allowing for combined analysis and examination of different implementation strategy components across varied contexts. Such findings, together with learnings exchanged between health promotion units, informed adaptions to local implementation strategies to improve their impact that were then subject to further improvement phases. To date, the LHS approach has generated more than a dozen RCTs across two phases (with a third phase ongoing), leading to iterative improvements in strategy effectiveness across diverse health promotion unit contexts.

## 4 CONCLUSION

LHSs hold considerable promise for the prevention of chronic disease. We offer here our thoughts and a framework to stimulate discussion about how this promise can become a reality. We acknowledge that our perspectives are borne from our experience in the Australian preventive health system. Their applicability, and that of the framework to LHSs similarly focused on chronic disease prevention program implementation in other contexts, warrants further consideration. In comparable high‐income countries, such as the United Kingdom (UK) and the United States, governments currently invest significantly in a number of initiatives to build health system capacity to facilitate the translation of evidence into health policy and practice. In the UK, these include the Collaborations for Leadership in Applied Health Research Centres, and Academic Health Science Centres.^87^ Given uncertainty regarding the impact of such centers,^88^ our LHS framework may provide some guidance as to how they may better support research generation and use by public health, chronic disease prevention services. Evaluation of efforts to do so would be particularly beneficial given the limited focus of investment in translation centers on the prevention of chronic disease.^49^ In low‐ and middle‐income countries, operationalizing LHS models may be more challenging. It will require innovative approaches that overcome resource constraints and adaptations to ensure application of the principles of the LHS framework are appropriate for the health system.

It is also important to acknowledge a range of ethical and administrative constraints associated with operating LHSs. While efforts to improve program or service implementation can occur within the context of routine service improvement activities, implementation trials typically meet the criteria of research and involve research participants. Consequently, these trials require institutional review board or ethics committee approval and oversight. Key ethical principles, including equipoise, must be upheld. We have discussed these issues relevant to implementation science in previous work.^40^ Additionally, for larger system‐level application of the LHS framework, as described in our second case study, dedicated resources for personnel to coordinate and undertake activities, and manage stakeholders are essential. This responsibility was undertaken by NCOIS with funding from an external grant. Sustainable LHS operations necessitate dedicated, long‐term resourcing for such activities, which we argue is a prudent investment for the health system. While there are many challenges to community organizations, prevention agencies, and the broader system operating as a LHS, we have demonstrated, at least locally, that these challenges are surmountable.

Larger scale LHSs focused on prevention operating across local, meso‐, and macro‐levels of the prevention have the greatest capacity to improve public health. Ideally, these would co‐ordinate the actions of organizations across levels, such that they are synergistic, efficient, and maximize effectiveness. Their realization will take leadership, skilled partnership, and considerable investments in resources and infrastructure. Much is to be learned from efforts to establish such systems of improvement. Given LHSs are an emergent model of health improvement, studying these experiences and shared learnings would significantly advance the field.

## FUNDING INFORMATION

Luke Wolfenden is supported by an NHMRC Investigator Grant (APP1197022). Nicole Nathan is supported by a Medical Research Future Fund Fellowship (APP1194785). Courtney Barnes and Cassandra Lane receive salary support from a NSW Ministry of Health PRSP Research Fellowship. Rebecca K. Hodder is supported by an NHMRC early career fellowship (APP1160419). Adam Shoesmith is support by a Hunter Medical Research Institute (HMRI) Population Health Research Fellowship. Rachel Sutherland is supported by a Medical Research Future Fund Fellowship (APP1194768) and a Hunter New England Clinical Research Fellowship.

## CONFLICT OF INTEREST STATEMENT

The authors declare no conflicts of interest.

## Supporting information


**Supplementary Figure 1.** Hypothetical example mapping the multiple organizations potentially involved in delivery of an evidence‐based intervention.

**Supplementary File 2.** Strategies to support policy agency organizational capacity to engage in implementation research and implementation research partnerships.
