# Supplementary material for: Learning health systems to implement chronic disease prevention programs: A novel framework and perspectives from an Australian health service
Source: Learn Health Syst. 2024 Oct 15;8(4):e10466. doi: 10.1002/lrh2.10466 (PMC11493556; doi:10.1002/lrh2.10466)
Supplement: Supplementary file 1 — Supplementary Figure 1. Hypothetical example mapping the multiple organizations potentially involved in delivery of an evidence‐based intervention. [file LRH2-8-e10466-s002.docx]

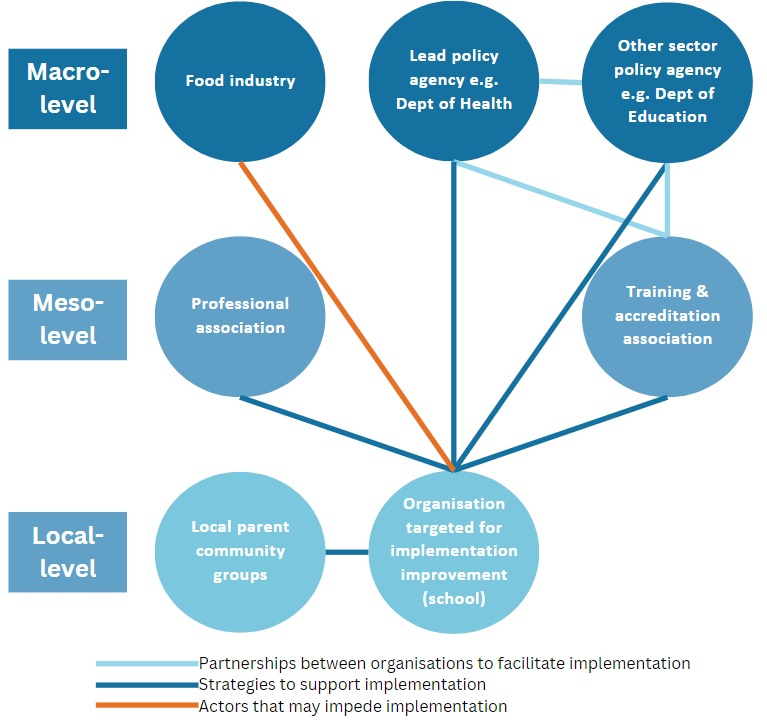


Figure 1. Hypothetical example mapping the multiple organisations potentially involved in delivery of an evidence-based intervention

Figure 1 characterises how such organisations may operate at different levels to support intervention delivery. It illustrates a hypothetical example of a government department of health (in this instance the lead agency) seeking to facilitate the implementation of a nutrition intervention in schools. It does this by providing implementation support direct to schools (e.g. resources and materials) and seeking to influence other organisations in the prevention system to do the same. In this example, to do the latter, it formed a cross sectoral macro-level partnership with an education agency (e.g. department of education); and a meso level partnership with a training and accreditation agency. These partnerships may be financial or non-financial and can be facilitated by the execution of partnership strategies (e.g. interagency meetings, written agreements; contracts). Ultimately such partnerships are designed to ensure the provision of comprehensive and co-ordinated support to facilitate implementation by schools - the targeted organisation - at the local level. Additionally, the figure illustrates that other organisations for which partnerships do not exist may also facilitate (e.g. supportive actions of professional association) or impede (e.g. actions of the food industry) the implementation of the school nutrition program.
