# Supplementary material for: Learning health systems to implement chronic disease prevention programs: A novel framework and perspectives from an Australian health service
Source: Learn Health Syst. 2024 Oct 15;8(4):e10466. doi: 10.1002/lrh2.10466 (PMC11493556; doi:10.1002/lrh2.10466)
Supplement: Supplementary file 2 — Strategies to support policy agency organizational capacity to engage in implementation research and implementation research partnerships. [file LRH2-8-e10466-s001.docx]

**Supplementary File 2: Strategies to support policy agency organisational capacity to engage in implementation research and implementation research partnerships**

Increasing the capability of the policy agency workforce can improve the likelihood that successful research partnerships will be cultivated to improve the implementation of health policies and programs. It may also increase their capacity to undertake research to address their own implementation evidence needs

The capacity building needs of policy agencies will depend on the ways in which they want to engage with implementation research and the research partnership they are seeking. Academic-controlled research will require limited workforce development strategies in policy agencies, such as training in accessing and critically appraising literature, and access to basic academic infrastructure, such as bibliographic databases, so that policy makers can identify, interpret and apply implementation research they find to address specific implementation evidence needs.

Models that involve co-creation will require organisational incentives and dedicated leadership to ensure purposeful involvement of the policy agency in the partnership.

Policy-led and policy-controlled partnerships require the highest levels of capacity building and support within the organisation, including the availability of essential research resources (such as statistical support, material resources and funding for data collection and dissemination of findings), dedicated research-practice roles, dedicated research time, as well as systems to facilitate networking and ongoing educational opportunities.

| Strategy | Examples | Academic-controlled | Academic-led | Co-creation | Policy-led | Policy-  controlled |
| --- | --- | --- | --- | --- | --- | --- |
| Research-trained and skilled staff within policy agency | Research-trained staff embedded/ integrated within policy agencies and governance positions |  |  |  | X | X |
|  | Research-practice roles |  |  |  | X | X |
|  | Funded/guaranteed research time |  |  | X | X | X |
|  | Secondments/joint position in policy and research institution |  |  |  | X | X |
|  | Co-location of staff and exchange of staff time |  |  | X | X | X |
| Provision of resources and infrastructure dedicated for research activity | Provision of, or access to, journal subscriptions | X | X | X | X | X |
|  | Available research space (desk, computer, software) |  |  |  | X | X |
|  | Statistical support |  |  |  | X | X |
|  | Data collection, management and infrastructure |  |  |  | X | X |
|  | Funding for research and dissemination of findings (conferences, open access publications) |  |  | X | X | X |
| Organisational incentives and rewards for undertaking research | Career advancement/promotion opportunities |  |  | X | X | X |
|  | Scholarships and paid research placements |  |  | X | X | X |
|  | PhD tuition support and scholarships |  |  | X | X | X |
|  | Awards, honours, public recognition of research excellence |  |  | X | X | X |
| Leadership commitment, involvement and accountability for research | Formal endorsement of research |  | X | X | X | X |
|  | Research leadership in position descriptions |  |  | X | X | X |
|  | Strategic plan with commitment to research activity/capacity |  |  | X | X | X |
|  | Building research questioning and evidence-based practice into service’s culture |  |  | X | X | X |
| Research training and capacity building | Online training, seminars, workshops, professional development, conferences | X | X | X | X | X |
|  | Local research champions/mentoring |  | X | X | X | X |
|  | Engagement in research projects, knowledge exchange, placements in research groups |  | X | X | X | X |
|  | Support to obtain formal qualifications/training |  | X | X | X | X |
|  | Observe-Act-Plan-Reflect used to encourage practitioners to initiate and lead research projects in their areas of interest |  |  |  | X | X |
| Networks and communication | Forums/conferences/multidisciplinary workshops and seminars aimed at building relationships and collaboration | X | X | X | X | X |
|  | Communities of practice |  |  | X | X |  |
|  | Joint PhD/student supervision between practice and academic institutions |  |  | X | X |  |
|  | Projects and technologies shared between collaborators |  | X | X | X |  |
| A formal research-practice entity and partnerships structures | Formal structures to enhance partnerships between universities and policy agencies to facilitate policy-led research (e.g. the Prevention Centre) |  | X | X | X |  |
|  | Contracting of research entities to provide research expertise, input and to undertake evaluations |  | X | X | X |  |
|  | Funding schemes aimed at supporting research partnerships (NHMRC Partnership Grants/CREs) |  | X | X | X |  |

This text and table is re-produced from:

Wolfenden L, Nathan N, Turon H, McCrabb S, Stickney B, Signy H, Rychetnik L, Crane M, Lee K, Pinheiro M, Sutherland R. Implementing policies and programs in prevention: Synthesis of knowledge from the The Australian Prevention Partnership Centre and CERI. Sydney, Australia: The Sax Institute, 2024.
